# Mindfulness-based stress reduction combined with early cardiac rehabilitation improves negative mood states and cardiac function in patients with acute myocardial infarction assisted with an intra-aortic balloon pump: a randomized controlled trial

**DOI:** 10.3389/fcvm.2023.1166157

**Published:** 2023-05-30

**Authors:** Kemei Wu, Miaomiao Wan, Huiqin Zhou, Cui Li, Xiaomin Zhou, E. Li, Ying Li, Chengwei Liu, Li Liu

**Affiliations:** ^1^ Division of Cardiac Care Unit, Department of Cardiology, Wuhan Asia Heart Hospital, Wuhan, China; ^2^ Department of Cardiac Rehabilitation, Wuhan Asia Heart Hospital, Wuhan, China

**Keywords:** acute myocardial infarction, intra-aortic balloon pump, cardiac rehabilitation, mindfulness-based stress reduction, anxiety, depression, profile of mood state, left ventricular ejection fraction

## Abstract

**Objective:**

To investigate the clinical effects of mindfulness-based stress reduction (MBSR) intervention combined with early cardiac rehabilitation (CR) on patients with acute myocardial infarction (AMI) assisted with an intra-aortic balloon pump (IABP).

**Methods:**

A total of 100 AMI patients with IABP assistance due to hemodynamic instability at Wuhan Asia Heart Hospital were enrolled in the study. The participants were divided into two groups using the random number table method (*n* = 50 each group). Patients receiving routine CR were assigned to the CR control group, while patients receiving MBSR plus CR were assigned to the MBSR intervention group. The intervention was performed twice a day until the removal of the IABP (5–7 days). Each patient's level of anxiety/depression and negative mood state were evaluated before and after intervention using the self-rating anxiety scale (SAS), self-rating depression scale (SDS), and profiles of mood state scale (POMS). The results of the control and intervention groups were compared. IABP-related complications and left ventricular ejection fraction (LVEF), measured with echocardiography, were also assessed and compared between the two groups.

**Results:**

The SAS, SDS, and POMS scores were lower in the MBSR intervention group than in the CR control group (*P* < 0.05). There were also less IABP-related complications in the MBSR intervention group. LVEF was significantly improved in both groups, but the degree of LVEF improvement was more significant in the MBSR intervention group than in the CR control group (*P* < 0.05).

**Conclusions:**

MBSR combined with early CR intervention can assist in alleviating anxiety, depression, and other negative mood states, reduce IABP-related complications, and further improve cardiac function in AMI patients with IABP assistance.

## Introduction

Patients with acute myocardial infarction (AMI) carry high in-hospital mortality if they present with hemodynamic instability (e.g., cardiac shock, cardiac arrest.) (1, 2). Currently, there are various mechanical circulatory devices such as cardiopulmonary bypass pumps/extracorporeal membrane oxygenation devices (ECMOs) and intra-aortic balloon pump (IABP)/non-IABP ventricular circulatory assist devices that are often used to help mitigate the adverse outcomes of cardiogenic shock until the underlying cause can be treated. Among the devices mentioned, the IABP is the most simple and cost-effective, as well as the easiest to implant and explant at the bedside by a cardiologist. It can also be effectively managed in an intensive care unit (ICU) by an intensivist, and therefore it is the widely accepted one in clinical practice (3). The IABP is a form of internal counterpulsation acting as an assistive circulatory support device through diastolic augmentation during inflation-enhancing coronary, cerebral, and systemic perfusion. Studies have indicated that the appropriate utility of an IABP in patients requiring hemodynamic support, either due to cardiogenic shock or risks of hemodynamic decompensation during a high-risk coronary intervention, significantly decreased in-hospital mortality, although this practice is still controversial in improving long-term prognosis (4–6). Patients with IABP assistance need to be positioned supine to keep the leg immobilized for 24 h post-removal if the femoral artery is the entry point for IABP insertion. However, long-term bed immobilization can lead to a deterioration of the patient's bodily functions and overall systemic decompensation (7, 8). At the same time, the impact of having the balloon in the body, in addition to the commotion in the ICU during the IABP assistance period, can easily cause negative emotions such as anxiety and depression. These negative influences were reflected by altered levels of biological markers such as increased concentrations of C-reactive protein, interleukin-6, tumor necrosis factor-α, and soluble interleukin-2 receptor (9). This course of treatment can have a large effect on the physical and psychological health of these patients (7, 8). Therefore, decreasing the negative effects of IABP implantation on patients’ mental health warrants further investigation.

Cardiac rehabilitation (CR) has been widely used in the clinic as an effective tool for secondary prevention after AMI. Its beneficial effects on short- and long-term mortality, hospitalization, and health-related quality of life have been well-established (10, 11), although there was a contradictory finding in an earlier study (12). Mindfulness-based stress reduction (MBSR) is a type of psychotherapy (meditation therapy) that was developed by Professor Jon Kabat-Zinn of the University of Massachusetts Medical Center in the 1970 s. It was originally designed for stress management (13) but is now being used to treat a variety of conditions, including mental illnesses, chronic pain, cancer, diabetes mellitus, hypertension, and autoimmune disorders (14–18). It works by encouraging the patients to do something positive for themselves, promoting non-judgmental acceptance and investigation of present experiences such as bodily sensations, internal mental states, thoughts, emotions, impulses, and memories. The goal is to maximize the relaxation of the body and mind, reduce suffering and distress, and increase general wellbeing (13, 19). Many clinical studies and meta-analysis have confirmed that the use of MBSR has a positive effect on many aspects of mental and physical health (14, 16, 20, 21). Negative psychological emotions such as anxiety and depression are common in patients with AMI, especially in those who are hospitalized in the ICU and/or depend on an assistive support device. Medical therapy with selective serotonin reuptake inhibitors (SSRIs) is usually prescribed, and it has proved effective in reducing depression symptoms among post-AMI patients (22). However, there has been no documented discussion of psychotherapy (such as MBSR therapy) on IABP implantation–related anxiety and depression. In this study, we observed the clinical effects of MBSR intervention, combined with early cardiac rehabilitation (CR), on post-AMI patients fitted with an IABP. Our results indicated that MBSR intervention, combined with early CR, could improve the negative mood states of these patients. This allows them to complete CR training more effectively and reduces IABP-related complications, further improving their cardiac function.

## Patients and methods

### Patients

**Figure 1:**
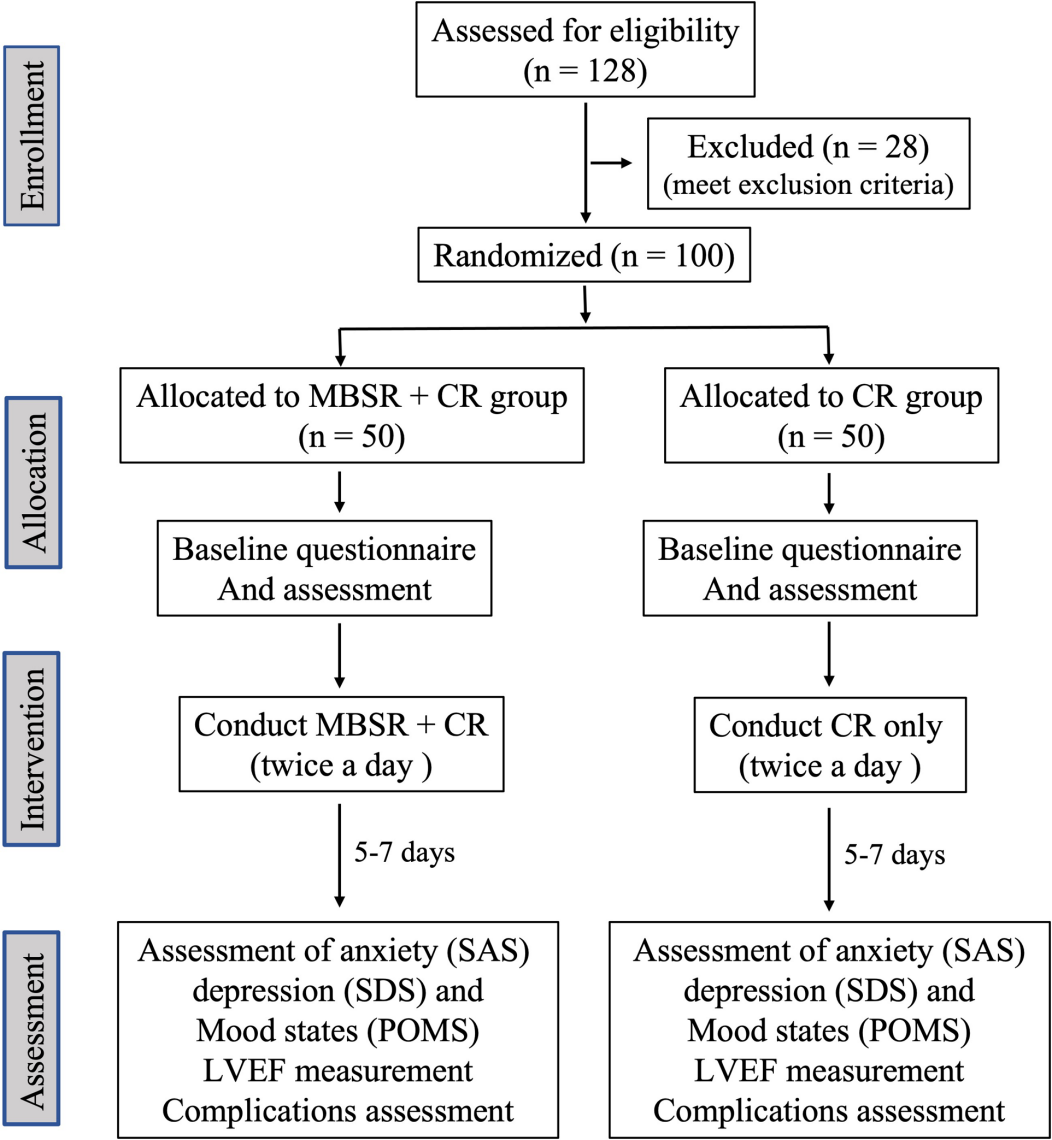
Figure consort flow diagram abbreviation: MBSR, mindfulness-based stress reduction; CR, cardiac rehabilitation; SAS, self-rating anxiety scale; SDS, self-rating depression scale; POMS, profiles of mood states; LVEF, left ventricular ejection fraction.

This was a single-center, randomized controlled trial designed for preintervention and postintervention assessments (the consort flow diagram shown as Figure 1). Patients were randomly assigned to either groups—MBSR + CR group or CR-only group—using computer-generated random tables. The patients were assessed on outcome measures (neuropsychological test, IABP-related complications, and cardiac function) before intervention and upon program completion (5–7 days).

Consecutive AMI patients who were implanted with an IABP from September 2021 to October 2022 in the Division of Cardiac Care Unit (CCU) of Wuhan Asia Heart Hospital were enrolled. The patients were then screened and excluded if they had any of the following: (1) abnormal cognitive function, visual/auditory impairment, intellectual disability/mental illness or consciousness disorders; (2) contraindications to cardiac rehabilitation intervention, such as previous thrombotic phlebitis or recent thromboembolism; (3) uncontrolled heart failure, severe atrial or ventricular arrhythmias, or obvious sinus tachycardia (>120 times/min); (4) acute systemic disease or fever, severe motor system abnormalities, and other metabolic abnormalities; (5) severe hepatic and renal diseases; (6) unwillingness or inability to cooperate with the study or receiving other psychological interventions; (7) patients with drug or alcohol dependence or psychoactive substance abuse; (8) and inability to accept psychological testing or having a history of other mental disorders. A total of 100 consecutive patients were included and divided into two groups using the random number table method: (1) the MBSR intervention group (*n* = 50): patients received MBSR intervention and routine CR, and (2) the CR control group (*n* = 50): patients received routine CR only. The baseline characteristics of the patients (e.g., age, gender, education levels, comorbidities, blood pressure), the percentage of the ST elevation myocardial infarction (STEMI)/non-STEMI, and the time of onset of AMI to hospital admission were obtained and compared between the two groups.

The study was approved by the Institutional Ethical Committee on Human Research of Wuhan Asia Heart Hospital (File number/Ethical clearance number: 2023-B010, SOP-LLWYH-025-03R). All patients gave written informed consent for participation in this study, use of blood samples, and provision of their clinical data.

### The cardiac rehabilitation (CR) and mindfulness intervention team

All enrolled patients received routine CR guidance from the CR team. The team consisted of the following persons: a chief physician responsible for assessment of the patient's condition and planning course of treatment, a professional rehabilitator responsible for CR training, a psychiatric nurse responsible for the assessment and collection of the questionnaires for anxiety, depression, and mood scales of the patients, and two additional nurses tasked with collecting and organizing the data of the enrolled patients. In addition to this team, there were two MBSR instructors who acted as psychological consultants and were responsible for mindfulness guidance and training for the patients in our study.

### MBSR intervention

Patients in the intervention group received MBSR therapy, led by a certified MBSR instructor, prior to routine CR (13, 23). The patients were first informed about the IABP, including the principle and therapeutic significance of IABP assistance, the location and depth of the tube insertion, and the importance of the coordinating body position. The instructor then began the practice of MBSR with each patient. The MBSR method is described as follows: the patients were first directed to lie flat on the bed. Using a soothing tone of voice, the instructor directed the patients to live in the present moment and embrace the psychological concepts of openness, acceptance, and non-judgment. The instructor then coached them to breathe slowly and deeply. The patients were instructed to place their hands on their abdomen as they inhaled slowly through the nose and exhaled slowly through the mouth, imagining their stomach expanding as a balloon. Afterward, the patients were instructed to feel the rise and fall of their stomach as they breathed, feeling their lungs fill with air. They were told to feel the relaxation of the body each time they breathed and to count each breath in gentle, continuous cycles. As they did so, the instructor then encouraged them to allow their heart to drift away with their breath, going back to the past and forward into the future, feeling the heart reach further before gently returning to the breath and back to the present under the prompting of the instructor. The patients were then asked to focus on their own quiet breathing and also to focus on “mindfulness” rather than “discomfort”. The patients practiced this exercise on their own until they were satisfied with their results, which usually took ∼30 min each time, twice per day. After this process came to an end, the rehabilitation therapist encouraged the patients to begin early CR training.

### CR training

Early CR training was carried out as follows: the instructor had the patient lie horizontally on the bed while the meaning, purpose, and requirements of the breathing exercise were relayed to them. Their head was raised at a 45°-degree angle, and they were instructed to perform progressive inhalation and exhalation training. Mouth-breathing trainers could be used to train or guide the patients with pursed-lip abdominal breathing. A vibration physical therapy device was used for pulmonary body therapy and effective cough guidance. The pressure of the device was set to 40 mmHg, with an interval of inflation of 4–8 s, twice a day, for 15 min each cycle. Once the patients began experiencing chest pain, palpitations, shortness of breath, dyspnea, or other clinical symptoms contraindicated by rehabilitation intervention, the rehabilitation training was stopped. The MBSR intervention and CR were performed until the removal of the IABP, which usually took 5–7 days.

### The evaluation of anxiety/depression, mood states, and assessment of IABP-related complications

Patients of both groups were evaluated on their anxiety, depression, and mood states before and 1 week after the CR or MBSR plus CR intervention by a team (including a psychiatrist and psychiatric nurse), whichwas blinded to the group allocation. This was done using the self-rating anxiety scale (SAS) for anxiety, the self-rating depression scale (SDS) for depression (24), and a short-form version of profiles of mood states (POMS) for mood states. POMS measures six different dimensions of mood swings, namely, tension or anxiety, anger or hostility, vigor or activity, fatigue or inertia, and depression or dejection (25). An SAS score of 50 points or more indicates anxiety, where 50–59 points is classified as mild anxiety, 60–69 points as moderate anxiety, and ≥70 points as severe anxiety. An SDS score of 53 or more indicates depression, where a score of 53–62 is classified as mild depression, 63–72 as moderate depression, and ≥73 as severe depression. The IABP-related complications (e.g., pulmonary infection, thromboembolism events, lower-extremity muscle atrophy) were also assessed at the same time.

### Evaluation of cardiac function by echocardiography

Cardiac systolic function (reflecting by left ventricular ejection fraction, LVEF) was evaluated with echocardiography before and after MBSR intervention on all enrolled patients by an echocardiographer who was blinded to the study group allocation using a Phillips Color Doppler Ultrasound Scanner (Phillips Medical System EPIC 7C, S5-1 probe). The optimal frame rate for 2D image acquisition is set between 60 and 100 frames per second. 2D grayscale images were acquired in the standard apical (apical 4 and apical 2 chambers) views, and 3 cardiac cycles were recorded. All images acquired and cardiac structure measurement methods were in accordance with the recommendations of the 2016 Chinese Adult Echocardiography Examination Measurement Guidelines (26). The two-plane Simpson method was used to calculate LVEF. The final LVEF was the average of apical 2 and apical 4 chambers.

### Statistics

Continuous variables are presented as the mean ± SD and were analyzed using independent-samples Student's *t*-tests. Categorical variables are presented as percentages or rates and were analyzed using the *χ*^2^ or Fisher’s exact test. Repeated measures ANOVA test was used to evaluate the differences of pre- and post-intervention between groups. A *P-*value of less than 0.05 was considered significant. All statistical analyses were performed using statistical software SPSS 25.0.

## Results

### The baseline characteristics of the MBSR group and the CR control group

When comparing the clinical data and patient demographics of the MBSR group with those of the CR control group, there were no observable statistical differences in age (*P* = 0.656), gender (*P* = 0.606), education levels, comorbidities, systolic blood pressure (*P* = 0.669), diastolic blood pressure (*P* = 0.352), the rate of STEMI (*P* = 0.539) and non-STEMI (*P* = 0.612), and the time between onset of AMI to arrival at the hospital (*P* = 0.826) (Table 1).

**Table 1:** Baseline characteristics of the MBSR group and the control group.

	MBSR group (*n* = 50)	Control group (*n* = 50)	χ^2^	*P*
Age (years)	59.72 ± 6.43	60.42 ± 7.12	0.516	0.607
Gender (%, Man)	74% (37)	70% (35)	0.198	0.676
BMI (kg/m^2^)	26.58 ± 3.51	26.11 ± 3.27	0.227	0.712
**Education**				
Below HS (*n*, %)	14 (28%)	15 (30%)	1.743	0.157
HS (*n*, %)	28 (56%)	25 (50%)	2.000	0.651
BS and above (*n*, %)	8 (16%)	10 (20%)	1.856	0.452
Systolic BP (mmHg)	135.38 ± 22.81	133.52 ± 20.47	0.429	0.669
Diastolic BP (mmHg)	69.56 ± 19.25	73.11 ± 18.73	0.925	0.352
**Comorbidities**				
Hypertension (*n*, %)	15 (30%)	18 (36%)	1.423	0.523
Diabetes (*n*, %)	17 (34%)	11 (22%)	2.000	0.157
Prior Stroke (*n*, %)	13 (26%)	9 (18%)	1.698	0.334
Smoking (*n*, %)	34 (68%)	33 (66%)	0.965	0.264
Hyperlipidemia (*n*, %)	29 (58%)	32 (64%)	1.137	0.886
**Acute Myocardial Infarction**				
STEMI (*n*, %)	29 (58%)	32 (64%)	0.378	0.539
Non-STEMI (*n*, %)	21 (42%)	18 (36%)	0.664	0.612
Time to hospital (h)	5.09 ± 2.89	5.21 ± 2.56	0.220	0.826

Values shown are means ± SD or percentages.

MBSR, mindfulness-based stress reduction; BMI, body mass index; HS, high school; BS, bachelor of science; STEMI, ST elevation myocardial infarction.

### MBSR intervention further improves anxiety and depression state in AMI patients assisted with IABP

During hospitalization at CCU, each patient in the MBSR + CR group received a total 10–14 sessions of MBSR. There was no significant difference in anxiety scores (SAS) and depression scores (SDS) between the two groups prior to MBSR intervention and CR training (*P* > 0.05). The levels of anxiety and depression were significantly decreased in both the control and the intervention groups (*P* < 0.05) following MBSR intervention and CR training. However, when compared with the CR control group, the MBSR group had lower SAS and SDS scores (*P* < 0.05) (Table 2).

**Table 2:** Comparison of anxiety and depression between the MBSR group and the control group.

	Group	Before	After	Interaction effect (*F*)	*P*	Effect size *n*^2^
Anxiety	MBSR group (*n* = 50)	63.60 ± 6.26	53.72 ± 4.40	72.923	<0.001	0.427
	Control group (*n* = 50)	63.04 ± 6.01	58.26 ± 5.52			
Depression	MBSR group (*n* = 50)	58.77 ± 3.69	48.09 ± 2.50	15.197	<0.001	0.134
	Control group (*n* = 50)	59.15 ± 3.25	52.62 ± 4.23			

Values shown are means ± SD.

MBSR, mindfulness-based stress reduction.

### MBSR intervention improves negative emotion in AMI patients assisted with IABP

Changes in mood states such as anger, fatigue, panic, nervousness, and depression were assessed using POMS-short-form. There was no significant difference in the POMS score of the two groups prior to intervention. However, a significant decrease in POMS scores (*P* < 0.05) was observed in the MBSR intervention group, while only the anger and nervousness scores were reduced in the CR control group (Table 3).

**Table 3:** Comparison of the brief mood state scale between the MBSR group and the control group.

	Group	Before	After	Interaction effect (F)	*P*	Effect size *n*^2^
Anger	MBSR group (*n* = 50)	10.62 ± 2.64	8.60 ± 2.23	29.073	<0.001	0.229
	Control group (*n* = 50)	10.80 ± 2.95	9.68 ± 2.53			
Fatigue	MBSR group (*n* = 50)	9.58 ± 1.65	6.92 ± 1.67	172.137	<0.001	0.637
	Control group (*n* = 50)	9.78 ± 1.94	8.78 ± 1.96			
Panic	MBSR group (*n* = 50)	7.94 ± 1.60	6.08 ± 1.35	21.535	<0.001	0.180
	MBSR group (*n* = 50)	8.06 ± 1.61	6.84 ± 1.58			
Nervous	MBSR group (*n* = 50)	10.54 ± 1.58	7.98 ± 1.81	15.858	<0.001	0.508
	Control group (*n* = 50)	10.68 ± 1.81	9.32 ± 1.97			
Depression	MBSR group (*n* = 50)	9.88 ± 1.30	7.88 ± 1.45	9.219	0.003	0.086
	Control group (*n* = 50)	9.96 ± 1.16	8.64 ± 1.12			

Values shown are means ± SD.

MBSR, mindfulness-based stress reduction.

### MBSR intervention improves cardiac function and reduces IABP-related complications in AMI patients assisted with IABP

LVEF was evaluated through echocardiography before and after MBSR intervention and CR training. The results showed that both CR and CR + MBSR can significantly improve LV systolic function, but the extent of improvement was more significant in the MBSR intervention group than in the CR control group (*P* = 0.001) (Table 4).

**Table 4:** The changes in cardiac function between the MBSR group and the control group.

	Group	Before	After	Interaction effect (F)	*P*	Effect size *n*^2^
LVEF (%)	MBSR group (*n* = 50)	38.34 ± 6.70	50.44 ± 9.76	87.618	<0.001	0.472
	Control group (*n* = 50)	39.79 ± 5.67	43.06 ± 6.75			

Values shown are means ± SD.

MBSR, mindfulness-based stress reduction; LVEF, left ventricular ejection fraction.

### MBSR intervention reduces IABP-related complications

No deaths were observed in either group, but IABP-related complications such as thromboembolism were much lower in the MBSR intervention group than in the CR control group (*P* = 0.031). No significant differences were observed on the occurrence of other complications such as lung infections, constipation, and lower-extremity muscle atrophy between the two groups (*P* > 0.05) (Table 5).

**Table 5:** Comparison of IABP-related complications between the MBSR group and the control group.

	Thromboembolic events	Lung infection	Constipation	Lower-extremity muscle atrophy
MBSR group (%) (*n* = 50)	1 (2%)	1 (2%)	2 (4%)	0 (0%)
Control group (%) (*n* = 50)	8 (16%)	3 (6%)	6 (12%)	2 (4%)
*F*	5.983	1.042	2.174	2.041
*P*	0.031	0.617	0.269	0.495

Values shown are means ± SD.

IABP, intra-aortic balloon pump; MBSR, mindfulness-based stress reduction.

## Discussion

As a significant, life-changing event, an AMI will inevitably bring about negative feelings such as anxiety and depression that could aggravate the patient's disease process (27). A meta-analysis of 23 hospital-based studies indicated that the prevalence of depression in hospitalized Chinese patients diagnosed with coronary heart disease was 51%, with 0.5%–25.4% classified as major depression (28). For AMI patients with cardiogenic shock or hemodynamic decompensation, some circulatory support devices such as an IABP are often used in place of aggressive pharmacological treatment. The use of these circulatory support devices typically instilled additional anxiety, depression, or other negative moods in post-AMI patients. Therefore, strategies to reduce negative mood states and any device-related complications in these patients become integral to effective treatment, warranting further investigation. CR is a multicomponent model of care, integrating exercise, patient education, and effective counseling. It is highly recommended for patients with AMI because of its proven benefits in reducing mortality and morbidity, alleviating anxiety/depression symptoms, and overall improvement of health-related quality of life (HRQoL) (10, 29–31). CR was routinely performed in our hospital for patients with AMI, and our results indicated that early implementation of CR after a diagnosis of AMI can significantly improve anxiety, depression, and other negative mood states in patients who have suffered from an AMI (Tables 2, 3).

Although initially developed for stress reduction and emotional management, MBSR has drawn increased interest in recent years because of its reliability in managing a variety of health-related disorders such as psychological distress, sleep disorders, chronic fatigue, pain management, psychiatric conditions, and cardiovascular health (14–18, 32). A recent study showed that it was also effective in dealing with physical and psychological symptoms caused by the long COVID syndrome (33). While its underlying mechanisms remain elusive, the four components of mindfulness have been proposed as its primary mode of action: attention regulation, body awareness, emotion regulation, and change in the perspective of self (34). These mechanisms are coupled with anatomical changes in the brain, such as a greater activation of the rostral anterior cingulate cortex and dorsal medial prefrontal cortex (35), greater cortical thickness (36), greater gray matter concentration in the right anterior insula (37), and decreased pain anticipation in the right parietal cortex and mid-cingulate cortex (38). Although MBSR has evolved to treat a variety of health-related disorders, limited data are available on the effects of MBSR on AMI patients receiving IABP assistance. Therefore, in this study, we explored whether MBSR intervention, combined with routine CR, can further alleviate anxiety, depression, other negative mood states, and IABP-related complications in this patient population of interest. Our results indicate that MBSR intervention, combined with early CR, can significantly alleviate patients' anxiety, depression, and other negative emotions while hospitalized in the CCU, as reflected by lower SAS, SDS, and POMS scores in the intervention group than in the control group. MBSR, combined with CR, can also reduce IABP-related complications, further improving cardiac function (LVEF) when compared with CR alone. Although the mechanisms underlying the improvement of cardiac function remain unclear, several studies indicated that the beneficial effects of MBSR on cardiac function might be associated with the reduction of stress (14, 15, 23), blood pressure (39), the improvement of heart rate variability, sympathovagal balance (40), and endothelial dysfunction (41). We have to point out that this is a single-center study, and therefore, potential selection bias cannot be excluded, and our findings may not apply to other medical centers. It is necessary to expand the study to multicenters in the future. In addition, our sample size is relatively small, and therefore, the study conclusion is relatively weak. A well-designed, larger-scale trial is warranted to confirm our conclusions. Finally, this study was designed as a short-term, observational study, and therefore, the long-term prognosis of patients receiving the MBSR intervention could not be readily assessed.

In summary, our study confirmed that MBSR intervention combined with early CR can alleviate anxiety, depression, and other negative moods in AMI patients receiving IABP assistance. It can also reduce IABP-related complications and further improve cardiac function. To the best of our knowledge, this was the first study investigating the effect of MBSR on negative mood states and cardiac function in AMI patients receiving IABP assistance while hospitalized in the CCU. However, it is necessary to further expand the clinical sample size and multicenters in future research.

## Data Availability

The original contributions presented in the study are included in the article, and further inquiries can be directed to the corresponding authors.
